# Co_3_O_4_ Nanoparticles Uniformly Dispersed in Rational Porous Carbon Nano-Boxes for Significantly Enhanced Electrocatalytic Detection of H_2_O_2_ Released from Living Cells

**DOI:** 10.3390/ijms23073799

**Published:** 2022-03-30

**Authors:** Lulu Xiong, Yuanyuan Zhang, Shiming Wu, Feng Chen, Lingli Lei, Ling Yu, Changming Li

**Affiliations:** 1 Key Laboratory of Luminescence Analysis and Molecular Sensing, Ministry of Education, Institute for Clean Energy and Advanced Materials, School of Materials and Energy, Southwest University, Chongqing 400715, China; luluxiong@email.swu.edu.com (L.X.); yuanyuanzhang55@163.com (Y.Z.); wsm19971206@email.swu.edu.cn (S.W.); cf199506@email.swu.edu.com (F.C.); leilingli@nsmc.edu.cn (L.L.); 2 Institute for Materials Science and Devices, School of Material Science and Engineering, Suzhou University of Science and Technology, Suzhou 215011, China; 3 Institute of Advanced Cross-Field Science and College of Life Science, Qingdao University, Qingdao 266071, China

**Keywords:** nano-boxes, tannic acid, Co_3_O_4_@CNBs, hydrogen peroxide sensor, electrochemical detection, living cell

## Abstract

A facile and ingenious method to chemical etching-coordinating a metal-organic framework (MOF) followed by an annealing treatment was proposed to prepare Co_3_O_4_ nanoparticles uniformly dispersed in rational porous carbon nano-boxes (Co_3_O_4_@CNBs), which was further used to detect H_2_O_2_ released from living cells. The Co_3_O_4_@CNBs H_2_O_2_ sensor delivers much higher sensitivity than non-etching/coordinating Co_3_O_4_, offering a limit of detection of 2.32 nM. The wide working range covers 10 nM-359 μM H_2_O_2_, while possessing good selectivity and excellent reproducibility. Moreover, this biosensor was used to successfully real-time detect H_2_O_2_ released from living cells, including both healthy and tumor cells. The excellent performance holds great promise for Co_3_O_4_@CNBs’s applications in electrochemical biomimetic sensing, particularly real-time monitor H_2_O_2_ released from living cells.

## 1. Introduction

H_2_O_2_ is a reactive oxygen species (ROS) frequently used as a marker for oxidative stress analysis. It is a by-product of reactions catalyzed by most oxidase enzymes [1], and is also involved in numerous physiological processes including cell differentiation and mediating immune responses [2,3]. Excess H_2_O_2_ will attack methionine residues and cysteine, which will cause cell damage and cytotoxicity. Owing to its peculiar capability, the concentration of H_2_O_2_ can be used as an indicator of several diseases diagnoses, such as Parkinson’s disease [4,5], cancer [6,7], diabetes [8] and acute myocardial infarction [9]. Thus, the determination of H_2_O_2_ is of great significance in biomedical, industrial, and academic applications. The H_2_O_2_ levels in the intracellular physiology range are from 0.001 to 0.7 μM [10]. Therefore, sensors with high sensitivity, specificity and broad working range are needed to probe the intracellular H_2_O_2_. The excellent detection of H_2_O_2_ mainly depends on the detection method and material two aspects. Among the technique for accurate and reliable detection of cellular H_2_O_2_, such as colorimetry [11,12], fluorescence [13,14], chromatography [15] and chemiluminescence [16], electrochemical techniques increasingly attracted attention due to their high sensitivity, good selectivity, low cost, as well as rapid response. For electrochemical detection, natural enzymes are usually the choice of sensing materials due to their remarkable specificity and high sensitivity in catalyzing the decomposition of H_2_O_2_. However, the inherent defect of natural enzymes, such as instability and ease to reduce or even deactivate the activity, limited their further applications [17]. Thus, non-enzymatic electrochemical sensors were proposed to overcome the limitations of natural enzyme sensing platforms [18]. Various nanomaterials have been used in H_2_O_2_ sensors, including transition metals oxides (e.g., Fe_3_O_4_, Co_3_O_4_, NiO, CuO) [19,20,21,22]. Transition metals have multiple oxidation states. They can absorb other substances onto their surface, meanwhile activating them in the process. These good abilities make them an excellent choice in synthesizing nanoenzymes [1]. Among these materials, Co_3_O_4,_ a kind of intrinsic p-type transition metal oxide, was reported in electrochemically detecting H_2_O_2_ because of its high electrochemical stability, fair price, and environmentally friendly [23]. However, the close-packed structures and poor electronic conductivity of Co_3_O_4_ could reduce their specific surface area and deteriorate its performance in H_2_O_2_ detection.

Metal-organic framework (MOF) possesses the periodic network structures made by the self-assembly of organic linkers and inorganic metal-containing nodes [24]. Recently, the unique merits of crystalline porous structure, highly dispersed metal components, and adjustable pore size of MOFs grant them outstanding performances in various applications [25]. In addition, MOF-derived carbon materials overcome the aggregation of metal nanoparticles that is induced by a further pyrolysis process [26]. Hence, metal-organic framework (MOF)-derived Co_3_O_4_ are promising in synthesis Co_3_O_4_ with uniform morphology and good electronic conductivity.

Tannic acid (TA) is a plant polyphenol. The chemical structure of TA is usually a decagalloyl glucose (C_76_H_52_O_46_) [27]. It widely exists in plant tissues such as tea, wood, and wine [28]. Its adhesive and reduction capability have been demonstrated in materials synthesis for lithium-ion batteries [29,30], dye remove [31], oil/water separation [32], catalytic [33], cell proliferation [34] and drug delivery [35]. As a kind of phenolic acid, TA is a weak organic acid and can release protons [36], which is applied in etching MOF materials to synthesize hollow structured materials [37].

In this study, to achieve sensitive and specific H_2_O_2_ detection, we rationally designed an ingenious method to synthesize ZIF-67 MOF-derived Co_3_O_4_ nanoparticles (NPs) dispersing in porous carbon nano-box (Co_3_O_4_ @CNBs) as a H_2_O_2_ nanozyme. The function of TA is to etch ZIF-67 while preserving the overall cubic architectures during thermal annealing process. The Co_3_O_4_ nanoparticles uniformly dispersed in porous carbon nano-boxes (Co_3_O_4_@CNBs) was synthesized by delicately tuning TA concentration and thermal annealing temperature. The sensing performance of Co_3_O_4_@CNBs in H_2_O_2_ sensing was characterized. The dispersion of Co_3_O_4_ NPs in the porous carbon nano-boxes (CNBs) was further investigated for its enhancement mechanism toward the specific reduction of H_2_O_2_. Moreover, the application of the Co_3_O_4_@CNBs H_2_O_2_ sensor was demonstrated in detecting H_2_O_2_ released from living cells.

## 2. Material and Methods

### 2.1. Chemicals

All of the chemicals were of analytical grade and used as received. The aqueous solutions were prepared with ultra-pure water (>18.25 MΩ/cm) obtained from Q-Grad^®^1 system (Millipore Corporation, Burlington, MA, USA). Cobalt nitrate hexahydrate (Co(NO_3_)_2_•6H_2_O), cetyltrimethylammonium bromide (CTAB), 2-methylimidazole (2-MeIm), tannic acid (TA), hydrogen peroxide (H_2_O_2_), glucose (Glu), cysteine (Cys), dopamine (DA), uric acid (UA), ascorbic acid (AA), glycine(Gly), sucrose (SUC), glutathione (GSH), urea and catalase from bovine liver were purchased from Aladdin (Shanghai, China). Phorbol 12-myristate-13-acetate (PMA) and Nafion (5%, wt %), were ordered from Sigma-Aldrich (Shanghai, China). Phosphate buffered saline (PBS, pH 7.4) was purchased from Dingguo (Beijing, China). Human non-small cell lung cancer A549, mouse breast cancer cells 4T1, and human umbilical vein endothelial HUVEC cell lines were obtained from the Type Culture Collection of the Chinese Academy of Sciences (Shanghai, China). Dulbecco’s Modified Eagle Medium (DMEM) medium, 10% fetal bovine serum (FBS), 1× antibiotic antimycotic were from Gibco (USA). Thiazolyl blue tetrazolium bromide (MTT) was purchased from Beyotime Biotechnology (Beijing, China).

### 2.2. Characterizations

The morphologies of the synthesized materials were observed by field emitted scanning electron microscopy (FESEM, JSM-7800 F, JEOL, Tokyo, Japan) and transmission electron microscopy (TEM, JEM-2100, JEOL, Japan). HAADF-STEM characterization was conducted with TEM (JEM-2100, JEOL, Japan). The surface properties of the materials were characterized by X-ray photoelectron spectroscopy (XPS, ESCALAB 250Xi, Thermo Scientific, Waltham, MA, USA). The crystal structure was characterized by X-ray diffraction (XRD, MAXima-X XRD-7000, Shimadzu, Tokyo, Japan). The chemical groups of the samples were recorded by Fourier transform infrared spectroscopy (FTIR, Thermo-Nicolet 6700, Thermo Scientific, MA, USA) with air as a reference. Thermogravimetric analysis (TGA, TA Instruments Q50, TA Instruments, New Castle, DE, USA) was performed using a thermal analyzer under airflow (10 °C min^−1^). JW-BK300C (JWGB SCI. & TECH., Beijing, China) determined N_2_ adsorption-desorption isotherms and pore-size distributions. All electrochemical measurements were performed at room temperature on a CHI 760D (Chenhua Instruments, Shanghai, China). PBS (0.01 M, pH = 7.4) was used as the electrolyte for all electrochemical measures except in detection with cell viability.

### 2.3. Preparation Co_3_O_4_@CNBs from ZIF-67

Synthesis of Co_3_O_4_@CNBs involves the following three-steps:

*Synthesis of ZIF-67 nanocubes (ZIF-67 NCs):* ZIF-67 NCs were synthesized according to the previous works [38]. 580 mg of Co(NO_3_)_2_•6H_2_O and 4 mg of etyltrimethylammonium bromide (CTAB) were dissolved in 20 mL of deionized water and marked as solution A. 9.08 g of 2-methyimidazole (2-MIM) was dissolved in 140 mL of deionized water, and marked as a solution B. Then the 20 mL solution A was rapidly injected into 140 mL solution B and stirred at room temperature for 20 min. The mixture was centrifuged at 10,000 rpm for 10 min. The collected precipitate (ZIF-67 NCs) was washed with ethanol several times and then dried in an oven at dried at 60 °C for 24 h.

*Synthesis of TA-Co nano boxes (TA-Co NBs)*: The as-prepared ZIF-67 NCs were first dispersed into 10 mL of ethanol, then poured into 150 mL of ethanol and deionized water mixture solution (Volume ratio of H_2_O and ethanol = 1:1) containing different concentration of TA solution (0 mg/mL, 0.5 mg/mL, 1 mg/mL, 2 mg/mL) and stirred at room temperature for 5 min. The precipitate collected by centrifugation was washed with ethanol and then dried in an oven at dried at 60 °C for 24 h. The TA etched ZIF-67 was recorded as TA-Co nano boxes (TA-Co NBs).

*Synthesis of Co_3_O_4_@CNBs*: The as-prepared TA-Co NBs powder was first annealed at 200 °C for 30 min and then further annealed at different temperatures (500 °C, 600 °C, 700 °C, 800 °C) for 1 h with a heating rate of 1 °C min^−1^ under N_2_ flow, and cooled down to room temperature naturally. After that, the powder was annealed at 200 °C for 6 h in air with a heating rate of 10 °C min^−1^. The obtained materials named as Co_3_O_4_@CNBs. In comparison, pristine ZIF-67 without TA etching was thermally annealed with the same condition and recorded as Co_3_O_4_@carbon (Co_3_O_4_@C).

### 2.4. Preparation of Co_3_O_4_@CNBs Modified Electrode

All electrochemical measurements were performed on a CHI 760D electrochemical workstation (Chenhua Instruments, China). A conventional three-electrode cell was used with a modified glass carbon electrode as the working electrode, Ag/AgCl (in saturated KCl solution) as the reference electrode, and platinum wire as the counter electrode. Glassy carbon electrodes (GCE) were polished with 0.3 and 0.05 μm alumina slurry on a polishing cloth and cleaned sequentially through water and ethanol under sonication for 3 min and dried in nitrogen flow for further use. Next, 7 μL 2 mg/mL Co_3_O_4_@CNBs aqueous dispersion was dropped on it and dried for 3 h at room temperature. After that, 5 μL 0.05% Nafion were dropped on it successively and dried at room temperature. Nafion film acts as a protective layer, preventing the falling of the loaded Co_3_O_4_@CNBs from the electrode. The supporting electrolyte of PBS (0.01 M, pH = 7.4) was deoxygenated using nitrogen before use and kept inside a nitrogen atmosphere. The prepared working electrodes were activated by cyclic voltammetric (CV) scanning for 20 cycles in the potential range from −1.0 to 1.0 V at a scan rate of 50 mV·s^−1^. Amperometric current-time curves (i-t) were collected at −0.22 V in 0.01 M 10 mL PBS by successive injecting H_2_O_2_ at 50 s intervals. 

### 2.5. Detection of H_2_O_2_ Released from Living Cells

In this work, three types of living human cells, A549, 4T1 and HUVEC cell were cultured in DMEM containing 10% FBS, 1 × antibiotic antimycotic. All the cells were supplemented with 10% FBS in a humidified incubator (with 5% CO_2_ atmosphere) at 37 °C and grown in polystyrene-coated T25 (25 cm^2^) cell culture flasks. Cells were washed three times with 0.01 M PBS (pH 7.4), detached by 1% Trypsin, collected by centrifugation, and the number was calculated using a cell counter. The response of H_2_O_2_ released from approximately 1.0 × 10^5^ cells was measured by Co_3_O_4_@CNBs modified GCE at −0.22 V in 2 mL DMEM medium.

## 3. Results and Discussion

### 3.1. Co_3_O_4_ NPs Dispersed in Porous Carbon Nano Boxes by TA Assisted Etchings

**Scheme 1:**
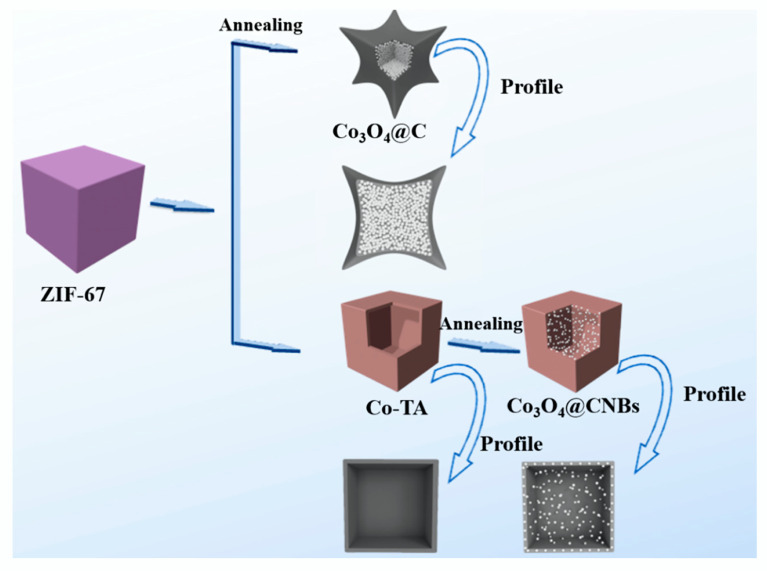
Schematic illustrations of the synthesis route of Co_3_O_4_@C and Co_3_O_4_@CNBs.

**Figure 1:**
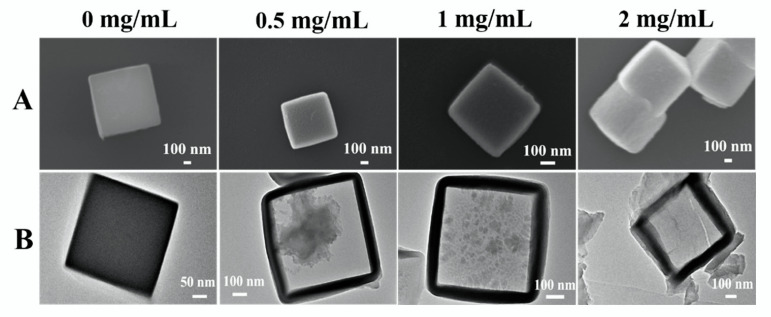
(**A**) FESEM images of TA-Co NBs with different TA concentration (0 mg/mL, 0.5 mg/mL, 1 mg/mL, 2 mg/mL); (**B**) Corresponding TEM images.

Synthesis of Co_3_O_4_@CNBs involves the following three-step reaction (Scheme 1). First, ZIF-67 was synthesized by using the co-precipitation method [38]. Next, TA was used to etch the ZIF-67 to form the unique Co_3_O_4_ NBs. Last, the Co_3_O_4_ NBs were thermal annealed to carbonize the TA and subsequent low-temperature oxidation in the air to form Co_3_O_4_@CNBs. The FESEM characterization found that the co-precipitation method synthesized ZIF-67 is uniform regular cubic with a smooth surface (Supplementary Information Figure S1). The size of the cubic is about 760 nm. In our approach, TA functions as green and facile etching agent to etch ZIF-67 directly without extra procedures and chemicals. We found that TA (0.5 mg/mL, 1.0 mg/mL, 2.0 mg/mL) treatment did not change the overall size and the surface morphology of the ZIF-67 cubic. As compared in in FESEM characterization (Figure 1A), TA treated ZIF-67 cubic has a size of 760 nm with a smooth surface. However, TEM characterization (Figure 1B) reveals that the cubic’s inner structure changes significantly after treated by TA with different concentrations. Though TA etched the inside of ZIF-67 cubic, the wall thickness of the cubic had no significant difference as TA concentration changed. The main effect of TA concentration influences the degree of etching reaction inside the cubic. As shown in Figure 1B, with the TA concentration increase from 0 to 2 mg/mL TA, the inside of ZIF-67 was solid at first and then showed the yolk-shelled heterogeneous structure. Finally, the ZIF-67 cubic was completely etched to form hollow interior TA-Co NBs (Figure 1B). Incubating ZIF-67 cubic in 2 mg/mL TA solution for 5 min resulted in a ZIF-67 NB with a wall thickness of about 80 nm.

The TA etching reaction is illustrated in Supplementary Information Figure S2. First, the protons released from TA etch the ZIF-67, releasing the Co^2+^ and 2-MIM simultaneously. At the same time, Co^2+^ and TA coordinate together quickly to form the TA-Co shell. The attached TA block the exposed surface of ZIF-67, thus protecting the outer parts of MOFs from further etching, resulting in internal etching of the ZIF-67 to form TA-Co NBs [29,37,39].

**Figure 2:**
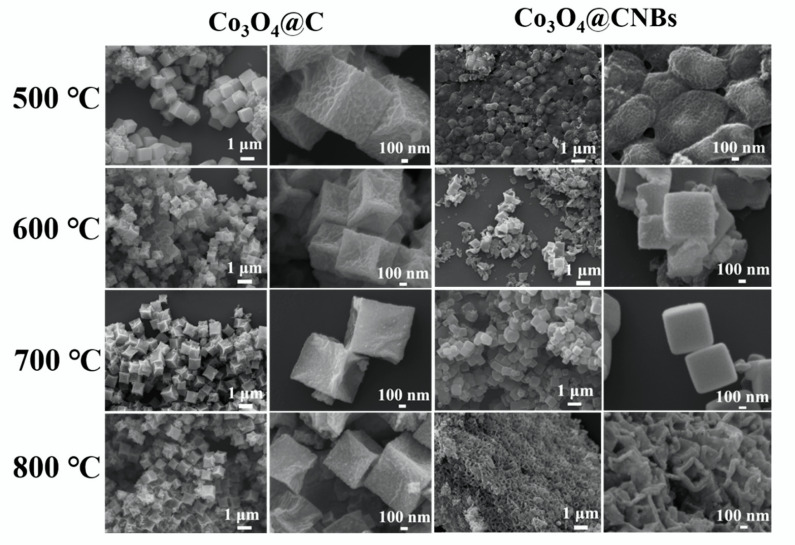
Co_3_O_4_@C: FESEM images of ZIF-67 after annealing at different temperature; Co_3_O_4_@CNBs: FESEM images of TA-Co after annealing at different temperature.

**Figure 3:**
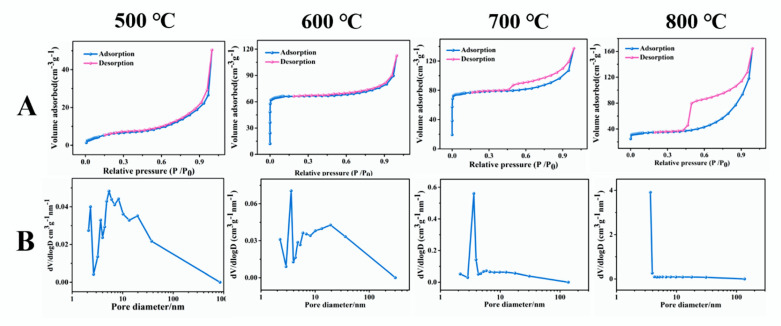
(**A**) N_2_ adsorption-desorption isotherms at different temperature; and (**B**) pore-size distribution plot at different temperature.

Next, TA-Co NBs were carbonized in an N_2_ atmosphere to synthesize the Co_3_O_4_@CNBs. First, the thermal carbonization and subsequent low-temperature oxidation in air at 200 °C were conducted with ZIF-67 cubic without TA treatment. From the SEM images in Figure 2, we found that even though the ZIF-67 is solid cubic, the thermal annealing still caused the shrink towards the inner side at the middle portion of each side. The morphology and structure have undergone apparent changes to a certain extent. This phenomenon is in line with previous studies in which ZIF-67 crystals obtained by direct annealing methods usually have a rough surface because of the aggregation of the nanoparticles [20,40,41,42]. However, as the FESEM images shown in Figure 2, the TA-Co NBs lost their structural integrity when the thermal carbonization was conducted at 500 °C, 600 °C, and 800 °C. Uniform cubic structures were observed from the products obtained at 700 °C. The SEM characterized morphology in Figure 2 confirms that TA-assisted etching successfully avoids the high-temperature carbonization induced cubic shrink. We speculated that the thermal carbonization caused structure changes could be induced by the partial collapse of pores on the nano-boxes. We examined the porosity of the products obtained from different temperatures. From the N_2_ adsorption-desorption isotherms curves shown in Figure 3, the porosity of Co_3_O_4_@CNBs obtained at 500, 600, 700, and 800 °C was 18.9 m^2^/g, 255.9 m^2^/g, 297.2 m^2^/g, 107.6 m^2^/g, respectively. The highest BET surface area is from the Co_3_O_4_@CNBs obtained at 700 °C. According to the N_2_ adsorption-desorption isotherms, the adsorption isotherm for 500 °C and 600 °C were similar to a BET type II isotherm. While 700 °C and 800 °C appears the BET type IV shape adsorption according to BET classification. It is worth noting that, as shown in Figure 2, materials at 800 °C has shown the collapse of the cubics. Collectively, the SEM (Figure 2) and BET results suggested that the intact cubic after annealing at 700 °C benefit the preserving of nano-pores on the nano box.

**Figure 4:**
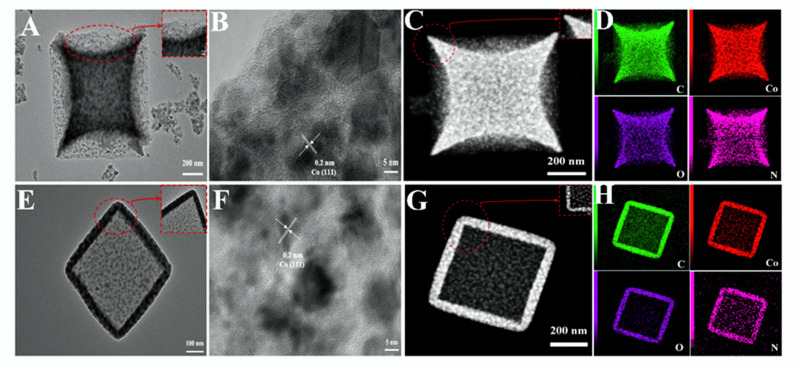
(**A**) TEM images of Co_3_O_4_@C; (**B**)HRTEM images of Co_3_O_4_@C; (**C**) HAADF-STEM images of Co_3_O_4_@C; (**D**) Elemental mapping images of C (green), Co (red), O (purple) and N (rose red) overlay of an individual Co_3_O_4_@C; (**E**) TEM images of Co_3_O_4_@CNBs; (**F**) HRTEM images of Co_3_O_4_@CNBs; (**G**) HAADF-STEM images of Co_3_O_4_@CNBs; (**H**) Elemental mapping images of C (green), Co (red), O (purple) and N (rose red) overlay of an individual Co_3_O_4_@CNBs.

The structures of Co_3_O_4_@C and Co_3_O_4_@CNBs were further compared through TEM characterization. Thermal annealing action caused the shrink of ZIF-67, resulting in a quadrangular star shape (Figure 4A). From the HRTEM characterization, the (111) planes of the metallic Co can be differentiated from the packed Co_3_O_4_ NPs (Figure 4B). The HAADF-STEM images (Figure 4C) and the corresponding elemental mapping images of C, Co, O, N elements in Co_3_O_4_@C (Figure 4D) clearly show the shrinking towards the inside at the four corners, and the inside of the cubic packed with dense and aggregated Co_3_O_4_ NPs. In comparison, with the action of TA, the dispersed Co_3_O_4_ was preserved nicely within the nano box (Figure 4E). HRTEM image of Co_3_O_4_@CNBs in Figure 4F shows the lattice fringe spacing is about 0.20 nm, corresponding to the (111) planes of Co_3_O_4_. The HAADF-STEM images of Co_3_O_4_@CNBs (Figure 4G) and the corresponding elemental mapping images of C, Co, O, N elements in Co_3_O_4_@CNBs (Figure 4H) confirmed that the Co_3_O_4_ NPs are highly dispersed in nano box. The evenly distributed C element would ensure electron transfer during electrochemical detection. The C, Co, O, N elements mapping images of Co_3_O_4_@CNBs thermal annealed at 500 °C, 600 °C, and 800 °C were shown in Supplementary Information Figure S3. From the FESEM and elements mapping, we confirmed that the C and Co are evenly distributed on the cubic. As the temperature increases, the structure gradually collapses at 800 °C.

**Figure 5:**
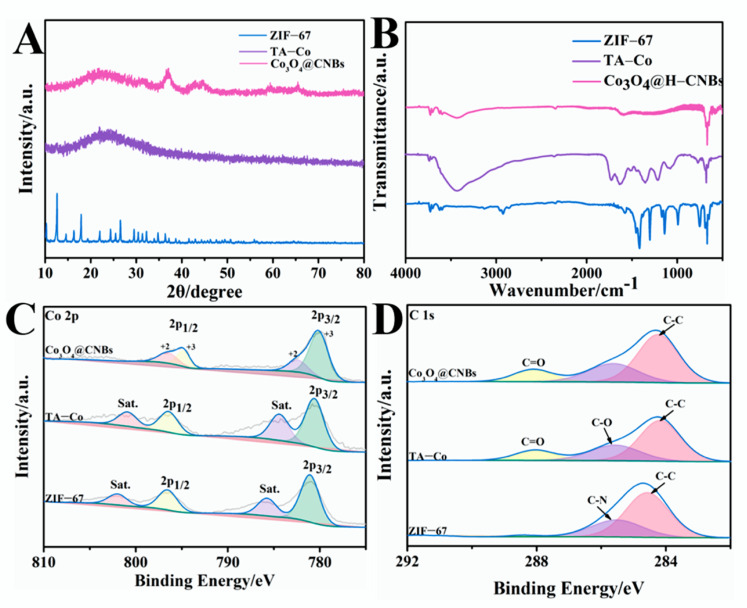
(**A**) XRD of ZIF−67, TA−Co and Co_3_O_4_@CNBs; (**B**) FTIR spectra of ZIF-67, TA−Co and Co_3_O_4_@CNBs; (**C**) High-resolution XPS spectra of C 1 s and (**D**) High-resolution XPS spectra of Co 2p.

Apart from tracking the reaction by morphological characterization (Figure 1, Figure 2, Figure 3 and Figure 4), the crystalline materials were characterized by XRD, FTIR, and XPS. First, the C 1s, Co 2p peaks can be observed from the XRD spectra, confirming the success in synthesis ZIF-67 (Figure 5A). The ZIF-67 precursor completely disappears after TA etching, indicating the completion of chemical transformation. Diffraction peaks of Co_3_O_4_@CNBs in XRD characterization perfectly match with the standard patterns of Co_3_O_4_ (PDF # 42-1467). The FTIR spectrum also supports the formation of Co_3_O_4_ (Figure 5B). FTIR spectrum shows that the prominent peaks at 3400 cm^−1^ are attributed to the vibration and stretching bands of functional groups of TA, which on account of TA complete substitution of 2-methylimidazole during the etching process [37]. Another strong bands at 667 cm^−1^ is attributed to the stretching vibration mode of Co-O with Co^2+^ [40]. 

XPS analysis was applied to reveal the elemental valence state of the Co_3_O_4_@CNBs. Compared with ZIF-67, TA-Co NBs present observable changes in C and Co’s contents, which are attributed to the introduction of TA and pyrolysis of organic ligands. As shown in Figure 5C, the spectrum of Co 2p can be best-fitted with two prominent peaks at binding energies by Co 2p_3/2_ and Co 2p_1/2_ peaks located at around 780.3 and 795.1 eV, corresponding to the state of Co_3_O_4_ phase. According to the XPS analysis (Figure 5D), the appearance of C=O, C-O, and C-C in a high-resolution spectrum of C 1 s are caused by the structure of TA [41]. As shown in Supplementary Information Figure S4, the TGA analysis reveals the weight content of Co_3_O_4_ in the composite is about 46.3 wt%. The weight loss under 250 °C is attributed to the evaporation of water molecules and air absorbed by the sample surface [42]. By analyzing FESEM, TEM, XRD, XPS, and FTIR results, we confirmed that Co_3_O_4_ nanoparticles well dispersed in Co_3_O_4_@CNBs synthesized from 2 mg/mL TA etching.

### 3.2. Dispersed Co_3_O_4_ NPs in Porous Carbon Nano Box Facilitate the Sensitive Electrochemical Detection of H_2_O_2_

**Figure 6:**
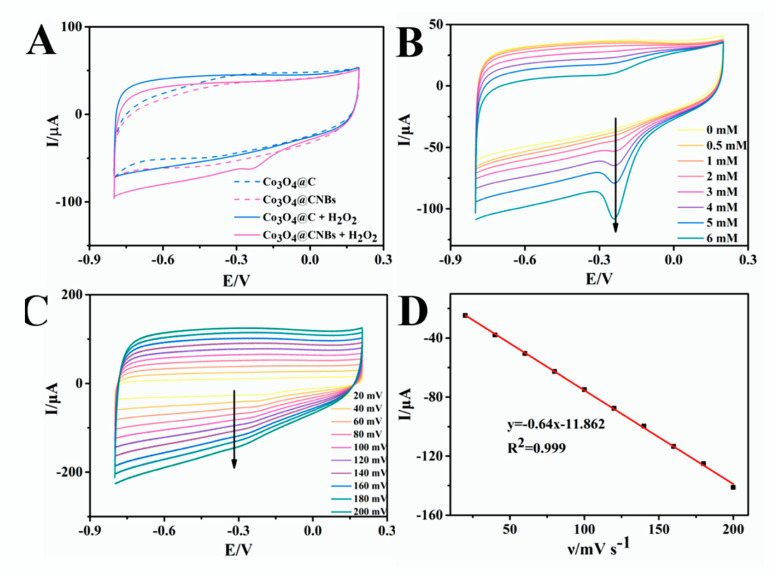
(**A**) CVs of Co_3_O_4_@CNBs/GCE and Co_3_O_4_@CNBs/GCE in the presence (purple line) and absence (blue line) of 2 mM H_2_O_2_ in 0.01 M PBS; (**B**) CVs of Co_3_O_4_@CNBs/GCE in the absence and presence of different concentrations (from 0 to 6 mM) of H_2_O_2_ in 0.01 M PBS; (**C**) CVs of Co_3_O_4_@CNBs/GCE in 0.01 M PBS at different scan rate (from 20 to 200 mV/s); (**D**) Linear relationship between the peak currents and the scan rates.

CV measurements were conducted to compare the electrochemical performance of carbonized ZIF-67 (Co_3_O_4_@C) and Co_3_O_4_@CNBs modified glassy carbon electrode (Co_3_O_4_@C/GCE and Co_3_O_4_@CNBs/GCE) in H_2_O_2_ detection. In Figure 6A, the dotted line and solid line represent the Co_3_O_4_@C/GCE and Co_3_O_4_@CNBs/GCE, respectively. The blue and purple lines represent the absence and addition of H_2_O_2_, respectively. With the addition of 2 mM H_2_O_2_, a cathode current around the potential of −0.22 V can be observed clearly from Co_3_O_4_@CNBs/GCE. In contrast, as shown in Figure 6A, no noticeable change was observed from Co_3_O_4_@C/GCE, indicating that Co_3_O_4_@C is inactive for electrooxidation of H_2_O_2_. Figure 6B displays the cyclic voltammetry (CV) curves of Co_3_O_4_@CNBs modified GCE in 10 mL 0.01 M PBS solution (pH 7.4) in the absence and presence of different concentrations of H_2_O_2_ (0.5, 1, 2, 3, 4, 5, and 6 mM). With the increasing concentrations of H_2_O_2_, a noticeable reduction peak current around −0.22 V dramatically increased. According to the previous reports, the electrocatalysis of H_2_O_2_ on the Co_3_O_4_@CNBs can be expressed by the following equation [43]:

$$2\mathrm{Co}\left(\mathrm{II}\right)+H_{2}O_{2}+H_{2}O\to 2\mathrm{Co}\left(\mathrm{III}\right)+2{\mathrm{OH}}^{-}+H_{2}O$$

The obvious reduction current indicated that Co_3_O_4_@CNBs nanocomposite have an excellent electrocatalytic activity for H_2_O_2_. The CV curves of Co_3_O_4_@CNBs/GCE were collected at different scan rates between −0.8 and 0.2 V in 0.01 M PBS (pH = 7.4). The reduction peak currents were enhanced with increasing scan rates. The current was in good linear with the scan rates (Figure 6C), suggesting that the H_2_O_2_ reduction on the Co_3_O_4_@CNBs/GCE’s surface was a typical adsorption control process.

**Scheme 2:**
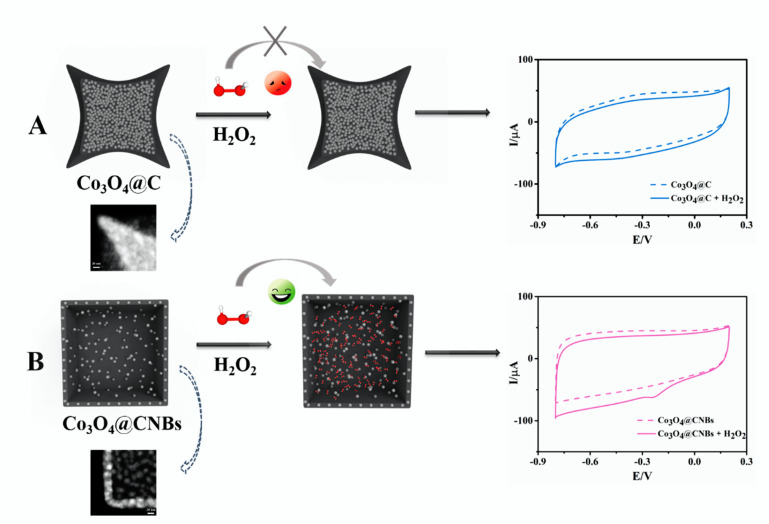
Schematic illustrations of the reaction mechanism of (**A**) Co_3_O_4_@C and (**B**) Co_3_O_4_@CNBs towards H_2_O_2_ sensing.

The different performance between Co_3_O_4_@C and Co_3_O_4_@CNBs towards H_2_O_2_ sensing is discussed. The thermal annealing and subsequent low-temperature oxidation will cause the four edges to shrink inward pristine ZIF-67 (Figure 4A,C). The structural changes are accompanied by the decrease of porosity (Supplementary Information Figure S5) because the porosity of Co_3_O_4_@C is 149.5 m^2^/g which is significantly smaller than that of Co_3_O_4_@CNBs (297.2 m^2^/g). Furthermore, the obvious aggregated Co_3_O_4_ nanoparticles in Co_3_O_4_@C (Figure 4A–C) impact the available sites of Co_3_O_4_ to react with H_2_O_2_ and potentially reduce the specific reaction area contributing to the electrochemical reduction of H_2_O_2_ (Scheme 2A). For the Co_3_O_4_@CNBs obtained from TA etching, the TA layer balanced the shrinkage stresses at different directions applied on the cubic during the annealing process. The architecture integrity avoids pore-collapse induced Co_3_O_4_ NPs aggregation (BET data in Figure 3 and TEM data in Figure 4). The porous structures would facilitate the transportation of H_2_O_2_ into the Co_3_O_4_@CNBs during electrochemical measurement. In addition, the TA protective layer alleviated the “stresses induced orientation contraction”, ensuring the uniform disperse of Co_3_O_4_ in CNBs to react with H_2_O_2_ (Scheme 2B).

### 3.3. Analytical Performance of Co_3_O_4_@CNBs Based H_2_O_2_ Sensors

To construct a sensitive H_2_O_2_ sensors, the electrochemical testing condition was optimized. The electrochemical behavior of Co_3_O_4_@CNBs/GCE was analyzed in 10 mL 0.01 M PBS (pH = 7.4). It is noted that the CV signal to H_2_O_2_ is affected by the concentration of Co_3_O_4_@CNBs and the adding volume. Supplementary Information Figure S6A,B show that 7 μL, 2 mg/mL Co_3_O_4_@CNBs leads to the highest signal. Hence, 7 μL 2 mg/mL Co_3_O_4_@CNBs were employed in the following study. The amperometric technique was employed to measure the response of Co_3_O_4_@CNBs modified electrode. The optimal working potential for detecting H_2_O_2_ was −0.22 V.

**Figure 7:**
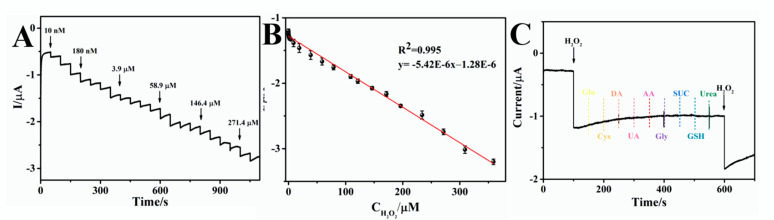
(**A**) Amperometric response of Co_3_O_4_@CNBs/GCE for different concentrations of H_2_O_2_ in 0.01 M PBS at an applied potential of −0.22 V; (**B**) The corresponding linear relation between the amperometric response and H_2_O_2_ concentration; (**C**) Amperometric response of Co_3_O_4_@CNBs/GCE for H_2_O_2_ in the occurrence of other substances.

With the optimized Co_3_O_4_@CNBs loading and electrochemical working voltage, the sensitivity and working range of the Co_3_O_4_@CNBs H_2_O_2_ sensor were characterized. The electrochemical response was recorded when successive adding varying H_2_O_2_ concentrations into 10 mL 0.01 M PBS (pH 7.4) solution. As shown in Figure 7A, the response was linear with H_2_O_2_ concentrations from 0.01 to 359 μM with a correlation coefficient of R^2^ = 0.995 (insert picture), and the regression equation was I (μA) = −5.42 × 10^−6^ − 1.28 × 10^−6^C (μM). The detection limit calculated was 2.32 nM (ratio of signal-to-noise (S/N) = 3). Comparison of Co_3_O_4_@CNBs based H_2_O_2_ detection with other H_2_O_2_ biosensors (Table 1) showed that our electrocatalytic performance of Co_3_O_4_@CNBs was satisfactory and even better than previous sensors.

Anti-interference ability is one of the critical analytical indicators for nonenzymatic biosensors. The amperometric method was adopted to study the interference of major interfering substances on Co_3_O_4_@CNBs based H_2_O_2_ detecting. As shown in Figure 7B, there are negligible signal responding to 0.15 mM glucose (Glu), cysteine (Cys), dopamine (DA), uric acid (UA), ascorbic acid (AA), glycine (Gly), sucrose (SUC), glutathione (GSH), and urea. While 0.05 mM H_2_O_2_ can induce a significantly larger signal, suggesting good selectivity for reducing H_2_O_2_. 

The stability and reproducibility of the Co_3_O_4_@CNBs-based sensor were also tested. Supplementary Information Figure S7 shows the amperometric electrochemical response of five modified independent electrodes from different batches. After statistical analysis of the test results, the relative standard deviation (RSD) obtained by five parallel tests was 2.4%, demonstrating a good reproducibility. The actual concentration of H_2_O_2_ present and the detected concentration were further tested, and the results in Supplementary Information Table S1 showed that the recovery rate of the Co_3_O_4_@CNBs-based sensor is from 95.62% to 105.78%. For the stability experiment, the modified electrode was stored at 4 °C for 15 days, and the current response to H_2_O_2_ (2 mM) was recorded every three days. It can be seen that after 15 days of storage, the current response of the sensor is maintained at 92% of the initial current.

### 3.4. Real-Time Detection of H_2_O_2_ Secreted from Living Cells by Co_3_O_4_@CNBs

**Figure 8:**
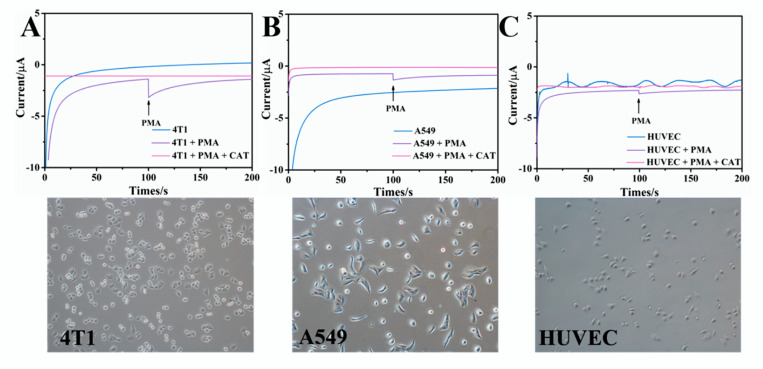
Amperometric response obtained at Co_3_O_4_@CNBs/GCE in the absence and presence upon addition of 2.5 μg/mL PMA and 20 μL 400 U·mL^−1^ catalase at −0.22 V. The insert is microscopy images of cells of (**A**) 4T1 cells; (**B**) A549 cells; (**C**) HUVEC cells.

To investigate the capability in actual samples application, Co_3_O_4_@CNBs H_2_O_2_ sensor was explored to real-time detect H_2_O_2_ from the living cell in a culture medium. The response of human epithelial cell HUVEC, mouse breast cancer cell 4T1, and human lung cancer cell A549 to PMA, a diester of phorbol, which can activate many cell types to produce H_2_O_2_, was studied. First, the potential cytotoxicity of Co_3_O_4_@CNBs was evaluated by the standard MTT assay. Supplementary Information Figure S8 reveals that no significant decrease in cell viability was observed from 10 to 50 μg·mL^−1^ Co_3_O_4_@CNBs-treated HUVEC cells and HeLa cells, demonstrating its good biocompatibility. The response of cells to PMA stimulation was measured by amperometric signal recorded in DMEM at −0.22 V. PMA is an activator widely used in in vitro experiments, which can stimulate cells to produce H_2_O_2_. As shown in Figure 8, the current has no obvious change when only cells exist. A promote and sharp increase of current peak was observed from all three cells challenged by 2.5 μg/mL PMA. In contrast, injecting PMA and catalase (CAT), an enzyme that catalyzes the decomposition of H_2_O_2_ into water and oxygen, at the same time will demolish the current change, which was observed in only PMA stimulation. Since CAT will decomposite the H_2_O_2_ released by PMA treated cells, by adding the PMA and CAT, we confirmed the current changes observed from cell stimulated by PMA only were induced by cell-released H_2_O_2_. The amperometric signal (Figure 8) recorded from the cells proves that the Co_3_O_4_@CNBs H_2_O_2_ sensor can detect the H_2_O_2_ released by living cells, highlighting its potential in studying cell metabolism. Next, the actual amount of H_2_O_2_ released from living cells was calculated according to the current and the calibration curve shown in Figure 7B. First, the current of the point reaching the plateau was read from the reaction curve. Then the H_2_O_2_ amount was calculated by placing the current value into the calibration curve equation. As shown in Figure 8, according to the current change and the calibration curve, the amount from three different cells was calculated at 0.16 μM (HUVEC), 0.26 μM (A549) and 0.19 μM (4T1), respectively. 

## 4. Conclusions

In conclusion, Co_3_O_4_@CNBs nanocomposites have been prepared using a facile and green method. Their application in the determination of H_2_O_2_ has been explored. The used TA improves the materials’ specific surface area and provides more active sites, further enhancing its electrocatalysis to reduce H_2_O_2_. The Co_3_O_4_@CNBs/GCE exhibits a good selectivity and high sensitivity for the determination of H_2_O_2_. Furthermore, the Co_3_O_4_@CNBs H_2_O_2_ sensor can detect the H_2_O_2_ secreted by HUVEC cells and 4T1, A549 cancer cells, highlighting its potential in biosensing and catalysis and biomedicine.

## Figures and Tables

**Table 1:** Comparison the sensing performance of Co_3_O_4_@CNBs reported with other H_2_O_2_ sensor in literatures.

Electrode Materials	Working Range/μM	Detection Limit/nM	Reference
Pt@Co_3_O_4_ NPs	10–300	100	[17]
Hollow Co_3_O_4_	0.4–2200	105	[20]
Au/Co@HNCF	25–2500	23	[44]
Au@C-Co_3_O_4_ NPs	-	19	[45]
Co_3_O_4_/NCNTs	5–11,000	1	[46]
Co_3_O_4_ NPs	-	21.7	[47]
Co_3_O_4_@H-CNBs	0.01–358.9	2.32	This work

## Data Availability

The data in the current study are available from the corresponding authors upon reasonable request.

## Supplementary Materials

The following supporting information can be downloaded at: https://www.mdpi.com/article/10.3390/ijms23073799/s1.
