# Supplementary material for: Co3O4 Nanoparticles Uniformly Dispersed in Rational Porous Carbon Nano-Boxes for Significantly Enhanced Electrocatalytic Detection of H2O2 Released from Living Cells
Source: Int J Mol Sci. 2022 Mar 30;23(7):3799. doi: 10.3390/ijms23073799 (PMC8999007; doi:10.3390/ijms23073799)
Supplement: Supplementary file 1 [file ijms-23-03799-s001.zip › ijms-1609328-supplementary.pdf]

Supporting Information

Co_3O_4 nanoparticles uniformly dispersed in rational porous carbon nano-boxes for significantly enhanced electrocatalytic detection of H_2O_2 released from living cells

Lulu Xiong [a], *Yuanyuan Zhang* [a], *Shiming Wu*[a], *Feng Chen*[a], *Lingli Lei*[a], *Ling Yu*[a*] *and Changming Li*[a,b,c *]

a. Key Laboratory of Luminescence Analysis and Molecular Sensing, Ministry of Education, Institute for Clean Energy and Advanced Materials, School of Materials and Energy, Southwest University, Chongqing 400715, P.R. China

b. Institute for Materials Science and Devices, School of Material Science and Engineering, Suzhou University of Science and Technology, Suzhou 215011, P.R. China

c. Institute of Advanced Cross-field Science and College of Life Science, Qingdao University, Qingdao, 266071, China

* Corresponding author: lingyu12@swu.edu.cn (L. Yu), ecmli@swu.edu.cn (C.M. Li)

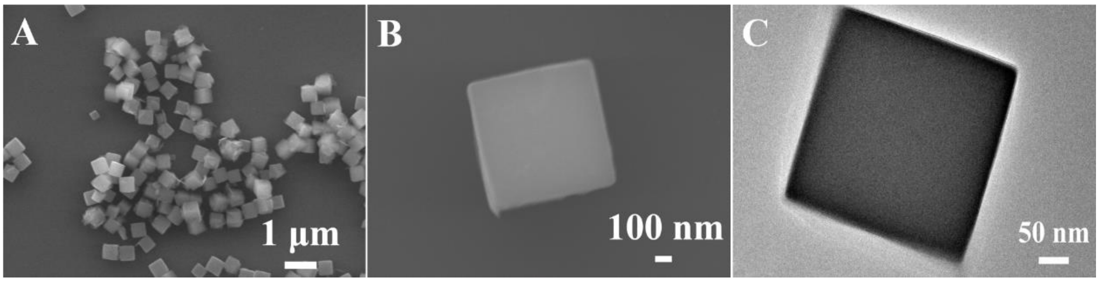

Figure S1 (A), (B) FESEM images of ZIF-67; (C) TEM images of ZIF-67.

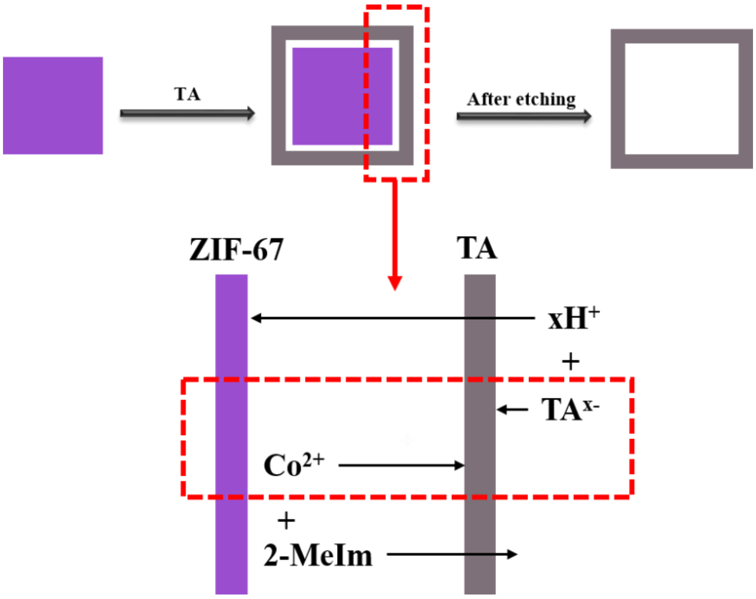

Figure S2 Schematic illustrations of the tannic acid (TA) based ZIF-67 etching.

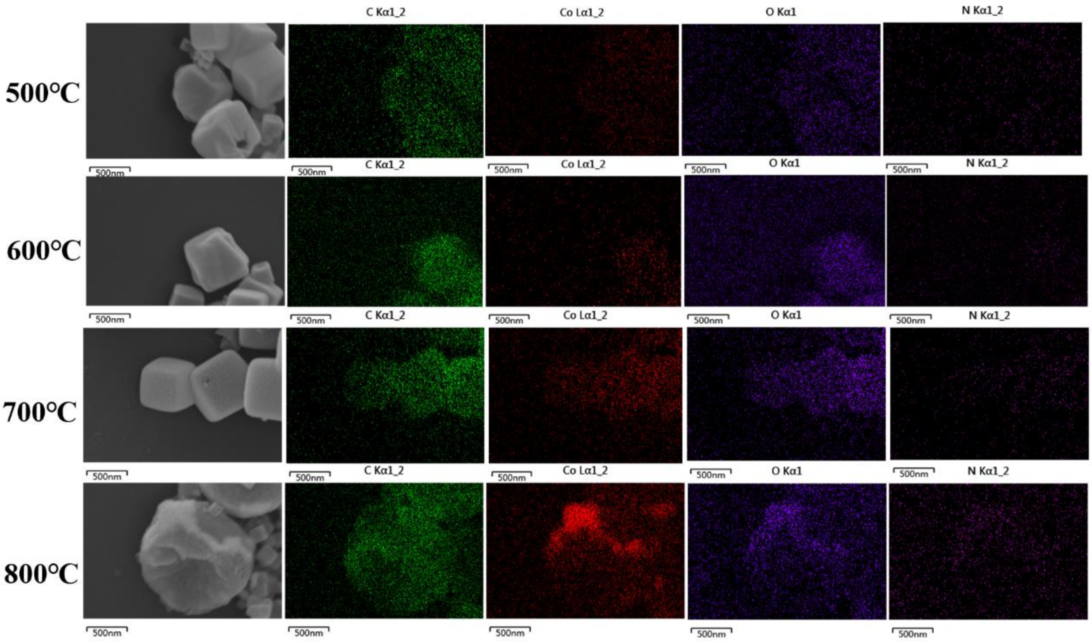

Figure S3. FESEM and elemental mapping images of C_3O_4@CNBs synthesized at different temperature: C (green), Co (red), O (purple) and N (rose red).

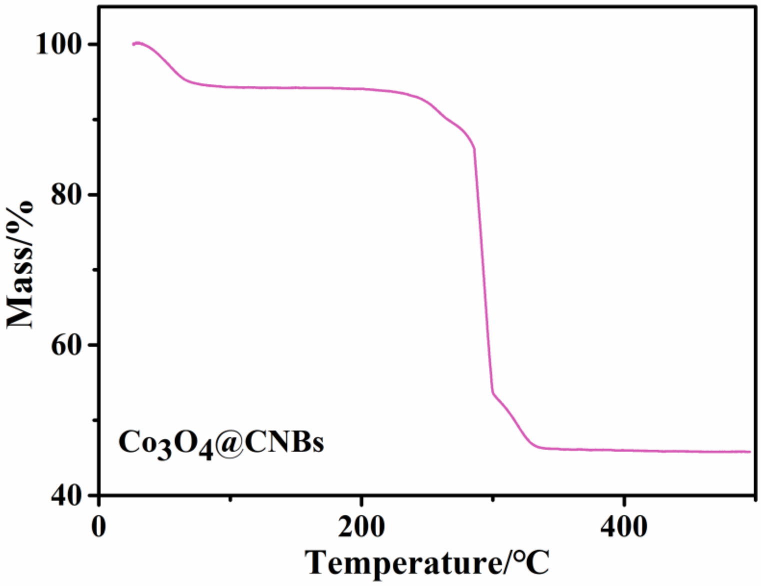

Figure S4 A TGA curve of Co_3O_4@CNBs in flowing air.

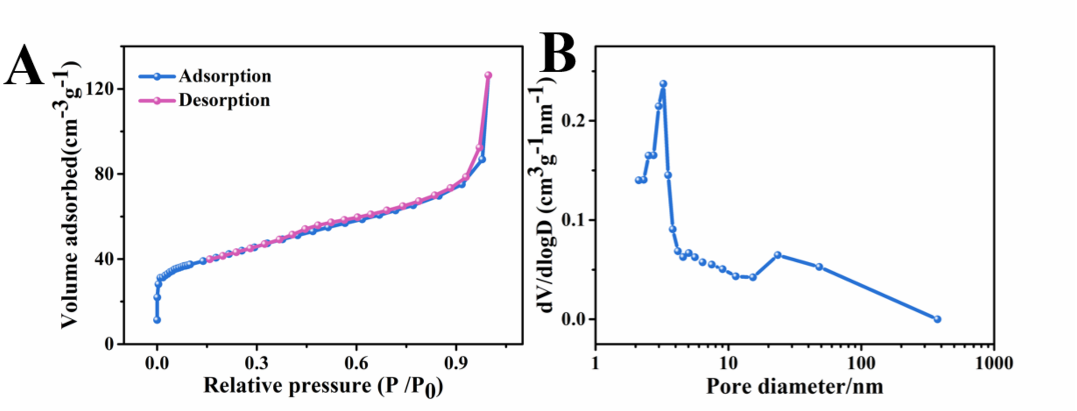

Figure S5 (A) N_2 adsorption-desorption isotherms and (B) pore-size distribution plot of Co_3O_4@C at 700 ℃.

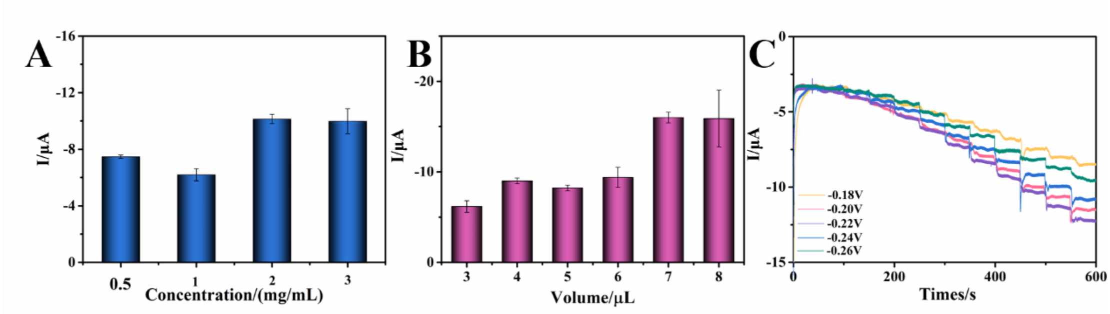

Figure S6 (A) Optimization study of Co_3O_4@CNBs concentration on the GCE; (B) Optimization study of volume of Co_3O_4@CNBs deposited on the GCE; (C) The amperometric i-t curves of Co_3O_4@CNBs/GCE for the successive additions of H_2O_2 at different operating voltages.

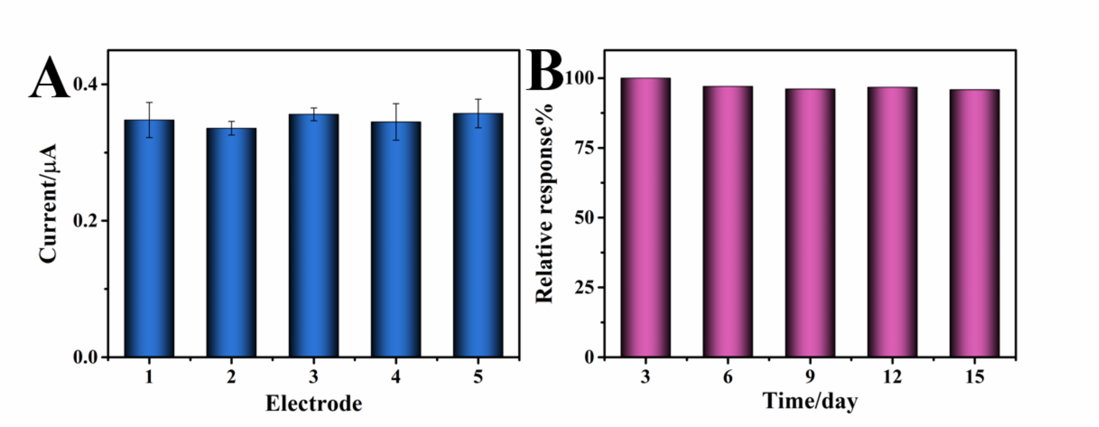

Figure S7 (A) Reproducibility between different Co_3O_4@CNBs/GCE; (B)Long-term storage stability of Co_3O_4@CNBs/GCE.

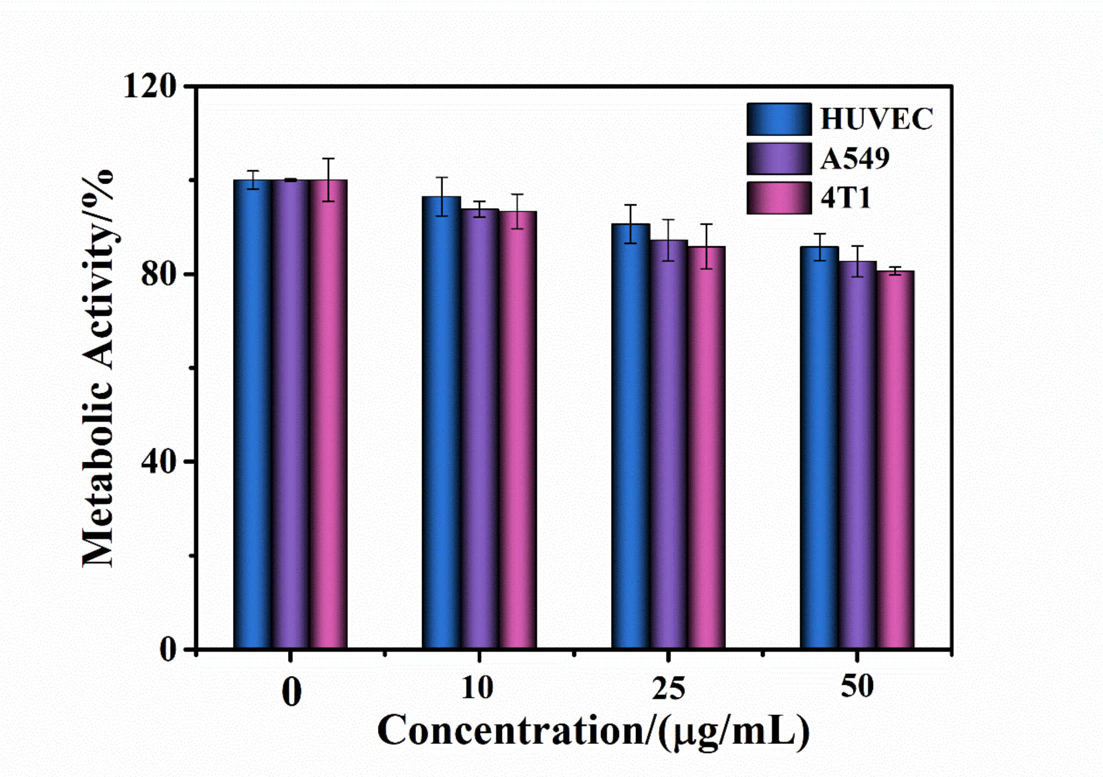

Figure S8. The cytotoxicity of Co_3O_4@CNBs on HUVEC, A549 and 4T1 cells characterized by cell metabolic activity.

Table S1. Recovery rate of the Co_3O_4@CNBs sensor in H_2O_2 testing.

Added Concentration (μM)	Mean Found(μM)	Mean recovery%
0.16	0.153	95.62
0.19	0.201	105.78
0.26	0.268	103.07
